# Characterizing the role of PP2A B’’ family subunits in mechanical stress response and plant development through calcium and ABA signaling in *Arabidopsis thaliana*

**DOI:** 10.1371/journal.pone.0313590

**Published:** 2024-11-14

**Authors:** Hyuk Sung Yoon, Kaien Fujino, Shenkui Liu, Tetsuo Takano, Daisuke Tsugama

**Affiliations:** 1 Asian Research Center for Bioresource and Environmental Sciences, Graduate School of Agricultural and Life Sciences, The University of Tokyo, Nishitokyo-shi, Tokyo, Japan; 2 Waksman Institute of Microbiology, Rutgers the State University of New Jersey, Piscataway, New Jersey, United States of America; 3 Laboratory of Crop Physiology, Research Faculty of Agriculture, Hokkaido University, Sapporo-shi, Hokkaido, Japan; 4 State Key Laboratory of Subtropical Silviculture, Zhejiang A & F University, Lin’an, Hangzhou, China; University of Delhi, INDIA

## Abstract

Protein phosphatase 2AB’’ (PP2A B’’) family subunits have calcium-binding EF-hand motifs, facilitating interaction with PP2A substrates. In *Arabidopsis thaliana*, the PP2A B’’ family subunits consist of six members, AtB’’α-ε and FASS. These subunits can interact with a basic leucine zipper transcription factor, VIP1, and its close homologs. Mechanical stress triggers PP2A-mediated dephosphorylation of VIP1 and its close homologs, leading to nuclear localization and gene upregulation to alleviate touch-induced root bending and leaf damage. However, the physiological roles of PP2A B’’ family subunits in the mechanical stress response in Arabidopsis remain unclear. This study aims to characterize such roles. A quadruple knockout mutant with T-DNA insertions in *AtB’’α*, *AtB’’β*, *AtB’’γ*, and *AtB’’δ* was generated. *atb’’αβγδ* mutants exhibited no significant damage upon brushing or touch-induced root bending compared to the wild type. Transcriptome analysis showed a significant decrease in the expression of *CYP707A3*, a gene potentially targeted by VIP1 that regulates abscisic acid (ABA) catabolism, in the *atb’’αβγδ* mutant compared to wild type leaves. However, other genes, including *XTH23*, *EXLA1*, and *CYP707A1*, also VIP1 targets, exhibited similar induction in both brushed *atb’’αβγδ* mutants and wild type leaves. We observed an enrichment of the CAMTA motif, CGCG(C/T) in the promoters of genes showing downregulated expression levels in brushed *atb’’αβγδ* leaves compared to brushed wild type leaves. These findings suggest that PP2A B’’ family subunits exhibit functional redundancy in the VIP1-dependent pathway but influence CAMTA-dependent gene expression under mechanical stress. Under calcium-deficient and ABA-supplemented conditions, growth of *atb’’αβγδ* seedlings was retarded when compared to wild type and single knockout mutants, *atb’’γ* and *atb’’δ*, indicating a crucial role in plant development by modulating calcium or ABA signaling.

## Introduction

Protein phosphatase 2A (PP2A) is a serine/threonine phosphatase consisting of three subunits: a scaffolding A subunit, a regulatory B subunit, and a catalytic C subunit [1, 2]. In *Arabidopsis thaliana*, there are 17 genes encoding PP2A B subunits, five genes for PP2A C subunits, and three genes for PP2A A subunits [1, 3]. The PP2A B subunits are classified into three families (B, B’, and B’’) based on their functional motifs, which play an essential role in determining the substrate specificity and functions of PP2A complexes [1]. This is consistent with the structural differences between these PP2A B subunit families: The PP2A B family B subunit has the WD40 domain, the PP2A B’ family B subunit has the B56 domain, and the PP2A B’’ family B subunit has EF-hand motifs that can bind calcium [1]. The Arabidopsis has six members of the PP2A B’’ family subunits: AtB’’α-ε and FASS (also known as TONNEAU2 (TON2) or EMBRYO DEFECTIVE 40 (EMB40)) [1, 4]. All PP2A B’’ family subunits, including AtB’’α-ε and FASS, interact with group I basic leucine zipper (bZIP) proteins, VIP1, and its close homolog, bZIP29, in a calcium-dependent manner [4–6]. They are responsible for the dephosphorylation of VIP1 [4, 6]. However, members of the PP2A B or PP2A B’ family cannot interact with VIP1 [6]. These results suggest that the different families of the PP2A B family subunits have different substrate specificities.

Under stable conditions, VIP1 and its homologs are localized in the cytoplasm [7]. However, when plant cells are exposed to mechanical stress and/or hypoosmotic stress, they are transiently localized to the nucleus [6, 8–13]. This nuclear localization of VIP1 and its homologs is dependent on calcium signaling [13] and PP2A-mediated dephosphorylation [4, 6]. VIP1 and its close homologs can upregulate genes that are responsible for mechanical stress tolerance and that contain the VIP1-binding element AGCTGG or AGCTGT (AGCTG(G/T)) in their promoters [8–10]. However, the single knockout of *AtB’’δ* and double knockout of *AtB’’β* and *AtB’’δ* do not affect the subcellular localization of VIP1 under hypo-osmotic stress conditions, possibly due to functional redundancy among the six members of the PP2A B’’ family subunits [4, 6].

Previous studies have revealed the involvement of *VIP1* and *RCN1*, a PP2A A subunit, in regulating root bending [10, 14, 15]. Overexpression of VIP1-SRDX, a variant of VIP1 fused with a synthetic repressor domain, results in enhanced root waving in plants grown on vertically oriented plates [10]. Co-expression of VIP1AAA, a variant with S → A mutations in three phosphorylation sites of VIP1, suppresses touch- and VIP1-SRDX-induced root bending [6]. The PP2A A subunit mutant *rcn1-6* also exhibits increased root bending under similar conditions [14]. PP2A B’’ family subunits can also interact with RCN1 in the presence of calcium to form a PP2A complex [4]. These findings raise the possibility that calcium-bound PP2A B’’ family subunits play a role in repressing root bending or enhancing mechanical stress resistance by recruiting PP2A A and C subunits to dephosphorylate VIP1. However, the precise role of PP2A B’’ family subunits in this process is largely unknown.

In this study, we aim to elucidate the physiological roles of PP2A B’’ family subunits in Arabidopsis by generating mutants with T-DNA insertions in the *AtB’’α*, *AtB’’β*, *AtB’’γ* and *AtB’’δ* genes. Our findings reveal that knocking out these genes impacts VIP1-mediated regulation of *CYP707A3* gene expression and influences function of calmodulin-binding transcription activators (CAMTAs). Furthermore, we found that PP2A B’’ family subunits are involved in plant development under calcium-deficient and ABA-supplemented conditions.

## Materials and methods

### Plant materials

The Arabidopsis ecotype Columbia-0 (Col-0) was used as the wild type control in all experiments. The seeds of *rcn1-6* (SALK_059903) [15], a line with a T-DNA insertion in *RCN1*, a PP2A A subunit gene, were obtained from the Arabidopsis Biological Resource Center (ABRC, https://abrc.osu.edu/). To generate the mutant with T-DNA insertions in PP2A B’’ family subunit genes, seeds of *atb’’α* (SALK_135978C, an *ATB’’α* knockout line), *atb’’β-1* (SAIL_1233_B02.V1, *ATB’’β* knockout), *atb’’γ* (SALK_032713C, *ATB’’γ* knockout), and *atb’’δ* (SALK_051304, *ATB’’δ* knockout) were obtained from ABRC. Seeds were sterilized by 10% (v/v) HClO solution with 0.05% (v/v) Tween 20, incubated at 4°C for 3 days and sown on the MS agar medium containing 0.5× MS salts, 0.8% (w/v) agar, 1% (w/v) sucrose, and 0.5 g l^–1^ MES, pH 5.8. Plants were grown at 22°C with the 16-h light/8-h dark photoperiod for 14 days on an agar medium and transferred on rockwool cubes as previously described to collect their seeds [13]. *atb’’β-1* and *atb’’γ* were crossed to generate the *atb’’β-1 atb’’γ* (*atb’’βγ*) double-knockout mutant. *atb’’α* and *atb’’δ* were crossed to generate the *atb’’α atb’’δ* (*atb’’αδ*) double-knockout mutant. The *atb’’β atb’’γ* mutant and the *atb’’α atb’’δ* mutant were crossed to generate the *atb’’α atb’’β atb’’γ atb’’δ* (hereafter *atb’’αβγδ*). T-DNA insertions in these mutant lines were confirmed by genomic PCR with KOD FX Neo (Toyobo) and primer pairs listed in S1 Table.

### Plant growth tests

To examine the root vertical growth indices (VGIs), we grew wild type, *atb’’αβγδ*, and *rcn1-6* plants on an agar medium (1.6% (w/v) agar, 0.5× MS salts, 1% (w/v) sucrose and 0.5 g l^-1^ MES, pH 5.8) tilted at a 75° angle, as previously described [6, 10, 13]. After five days, the seedlings were photographed, and the lengths of their primary roots and the vertical projections from these roots were measured using ImageJ software [16]. To calculate the root VGIs, the vertical projection lengths were divided by the primary root lengths, as described previously [17].

To examine plant responses to mechanical stress, the wild type, *atb’’αβγδ* and *rcn1-6* plants were grown on the agar medium as described above in the ’’Plant materials’’ subsection. 14-day-old plants were transferred to soil on pots with a 4 cm × 4 cm size. Those plants were further grown for a week on the soil with the 4 plants/pot planting density, and rosette leaves of resulting 21-day-old plants were brushed for 10 sec twice a day, at 9:00 and 16:00 as described previously [18]. This brushing treatment was applied to the same plants every day for another six days. To examine survival rates, plants with emerging green leaves were counted as survivors 7 days after the final brushing treatment was applied.

To examine plant responses to calcium deficiency, wild type, *atb’’γ*, *atb’’δ* and *atb’’αβγδ* plants were grown on the MS agar medium lacking CaCl_2_. To assess plant phenotypes in response to ABA and abiotic stresses, the same plant lines were cultivated on MS agar medium supplemented with 1 μM abscisic acid (ABA), 100 mM NaCl, or 200 mM mannitol. 14-day-old seedlings were utilized to measure both cotyledon lengths [9] and greening rates [19]. The cotyledon lengths were measured from the tip of the leaf to the hypocotyl using ImageJ software. The greening rates were determined based on the number of plants that exhibited emerging green cotyledons [19].

### RNA-seq

Rosette leaves from 21-day-old wild type and *atb"αβγδ* plants were subjected to the brushing treatment for three days, as described above in the "Plant growth tests" subsection. The leaf samples were collected 20 minutes after the final brushing treatment was applied. Total RNA was extracted from these leaves using the NucleoSpin RNA Plant kit (Macherey-Nagel). The brushing treatment and RNA extraction procedures were replicated three times for each genotype, providing three biological replicates. For RNA-Seq library preparation and runs, the extracted RNA samples were sent to Novogene Bioinformatics Institute (Beijing, China) and sequenced using the NovaSeq 6000 platform (Illumina, Inc., San Diego, CA). The resulting read data was deposited in the Sequence Read Archive (SRA) of the National Center for Biotechnology Information (NCBI) under the BioProject accession number PRJNA899989.

The clean reads were aligned to the Arabidopsis reference genome sequence (Araport11) obtained from The Arabidopsis Information Resource (TAIR, https://www.arabidopsis.org/) using HISAT2 [20]. The mapping results were then processed with featureCounts [21] to determine the counts of reads mapped to each mRNA region. These counts were converted to transcripts per million (TPM) values by a custom Perl script. All TPM values in this study are provided in S1 Dataset. Independently of this TPM analysis, fold changes of mRNA levels between different samples and associated false-discovery rate (FDR)-based adjusted *P* values were obtained by DESeq2 [22]. mRNA was regarded as differentially expressed mRNA (or a differentially expressed gene, DEG) when its adjusted *P* value was smaller than 0.05. To identify enriched motifs within the promoters of the DEGs, 500-bp upstream sequences from the start codons of the DEGs were obtained as their promoter sequences from TAIR. All the detected motifs are shown in S2 Dataset.

Motif analysis of these promoter sequences was performed using HOMER (v. 4.11), with the 500-bp promoter sequences of 2000 randomly selected Arabidopsis genes as the background. All the detected motifs are shown in S3 Dataset.

### Quantitative RT-PCR (qRT-PCR)

For total RNA samples, both the wild type and *atb"αβγδ* leaves were subjected to brushing treatment as described in the "RNA-Seq" subsection above. The ReverTra Ace reverse transcriptase (Toyobo) was used with the oligo (dT) 15 primer to synthesize cDNA from 1 μg of the isolated total RNA. Subsequently, the resulting cDNA solutions were diluted using distilled water and used as templates. For quantitative PCR, a mixture of the templates, GoTaq qPCR Master Mix (Promega, Fitchburg, WI, USA), and the primers listed in S2 Table were used. The PCR was performed using the StepOne Real-Time PCR System (Thermo Fisher Scientific, Waltham, MA, USA). To determine the relative expression levels, we applied the comparative cycle threshold method, using *UBQ5* as the internal control gene.

### *In-vitro* dephosphorylation assays

The pGBK-AtBβ and pGBK-AtB’’δ constructs were prepared as the previously described [6]. To obtain Myc-tagged versions of AtBβ and AtB’’δ, an *in vitro* transcription-translation assay was performed using the TNT Quick Master Mix (Promega, Madison, WI, USA) and the corresponding pGBK-AtBβ and pGBK-AtB’’δ constructs. To confirm the presence of Myc-tagged proteins, we used Anti-Myc-tag mAb-Magnetic Beads (Medical & Biological Laboratories (MBL) Co., Ltd., Tokyo, Japan). The beads were added to the protein solutions and gently incubated at room temperature with continuous shaking for one hour. Subsequently, the beads were washed three times with Tris-buffered saline with Tween-20 (TBST: 150 mM NaCl and 20 mM Tris-HCl, pH 7.5 with 0.1% (v/v) Tween-20), followed by treatment with 2× SDS sample buffer at 100°C for 5 minutes. The resulting supernatants were subjected to SDS-PAGE. For western blotting, an anti-Myc-tag pAb (MBL) was used as the primary antibody, and then Anti-Rabbit IgG (H+L-chain) (MBL) was used as the secondary antibody. The signals derived from the Myc tag were detected using SuperSignal West Pico Chemiluminescent Substrate and ImageQuant LAS 4000 mini.

For the *in vitro* dephosphorylation assays, glutathione S-transferase (GST), GST-fused VIP1 (GST-VIP1), maltose-binding protein (MBP), and MBP-fused CALCIUM-DEPENDENT PROTEIN KINASE 21 (MBP-CPK21) were expressed in *Escherichia coli* cells as previously described [4, 6]. The presence of MBP-fused proteins was confirmed by western blotting using an anti-MBP monoclonal antibody (New England Biolabs) and an anti-IgG (H+L chain) (mouse) pAb-HRP (Medical & Biological Laboratories Co., Ltd, Tokyo, Japan) (S1A Fig). Subsequently, we purified GST and GST-VIP1 using Glutathione Sepharose 4B resin according to the manufacturer’s instructions. The resin containing either GST or GST-VIP1 was mixed with a solution of purified MBP or MBP-CPK21, and the mixture was incubated at room temperature for one hour. After incubation, the resin was washed three times with TBST. The resulting resin was then mixed with a solution of Myc-AtBβ or Myc-AtB’’δ, along with 10 mM CaCl_2_ and 1 mM MnCl_2_, in the presence of the TNT Quick Master Mix. This mixture was incubated at room temperature for one hour. Subsequently, the resin was washed three times with TBST, resuspended in 2× SDS sample buffer, and incubated at 100°C for 5 minutes. The resulting supernatants were subjected to SDS-PAGE. The phosphorylated proteins were detected using western blotting with Phos-tag biotin BTL-104 (Fujifilm Wako, Osaka, Japan) and HRP-Conjugated Streptavidin. Furthermore, the membranes were incubated in a Ponceau S staining solution and washed with water to visualize GST and GST-VIP1 proteins on the membranes.

To further examine AtB’’δ-dependent VIP1 dephosphorylation, we used cantharidin, a PP2A inhibitor. GST-VIP1 was phosphorylated using MBP-CPK21 and then subjected to a reaction with either Myc or Myc-AtB’’δ in the presence or absence of 50 μM cantharidin. The phosphorylation-derived signals were detected through western blotting as described above. To quantify the level of protein phosphorylation in GST-VIP1, the Phos-tag biotin-derived signals were measured using ImageJ software. These values were expressed as relative values by normalizing them with the Phos-tag biotin-derived signals of GST-VIP1 incubated with Myc alone, which was considered as the reference (assigned a value of 1). The resulting values represent the level of protein phosphorylation. The presented results in the figure are the mean values and standard deviation (SD) obtained from three independent replicates.

### Accession numbers

AT1G43700 (*VIP1*), AT1G17720 (*ATBβ*), AT5G44090 (*AtB’’α*), AT1G03960 (*AtB’’β*) AT1G54450 (*AtB’’γ*), AT5G28900 (*AtB’’δ*), AT4G04720 (*CPK21*), AT1G25490 (*RCN1*), AT3G45970 (*EXLA1*), AT4G25810 (*XTH23*), AT4G19230 (*CYP707A1*), AT5G45340 (*CYP707A3*), and AT3G62250 (*UBQ5*).

## Results

### PP2A B’’ family subunits are responsible for VIP1 dephosphorylation

VIP1 interacts with six members of the PP2A B’’ family subunits but not with other PP2A B family subunits [6]. VIP1 is phosphorylated by CPK21 protein kinases [4, 6], and its dephosphorylation is dependent on PP2A B’’ family subunits [4]. To examine whether another PP2A B family subunit, AtBβ, can induce VIP1 dephosphorylation, Myc-tagged forms of AtBβ (Myc-AtBβ) and AtB’’δ (Myc-AtB’’δ) were expressed through coupled transcription-translation in rabbit reticulocyte lysate-based solutions (S1A Fig). When GST-VIP1 phosphorylated by MBP-CPK21 was incubated with Myc-AtBβ and rabbit reticulocyte lysates, the western blotting signal for phosphorylated GST-VIP1 using Phos-tag biotin did not decrease (S1B Fig). In contrast, the phosphorylation signal of GST-VIP1 decreased in the presence of Myc-AtB’’δ and rabbit reticulocyte lysates (S1B Fig). Furthermore, Myc-AtB’’δ-mediated GST-VIP1 dephosphorylation was inhibited when the reaction mixture contained cantharidin, a PP2A inhibitor (S1C Fig). These results indicate that PP2A B’’ family subunits, rather than other PP2A B family subunits, are responsible for the dephosphorylation of VIP1.

### A quadruple knockout mutant with T-DNA insertions in *AtB’’α*, *AtB’’β*, *AtB’’γ*, and *AtB’’δ* does not affect responses to mechanical stress

**Fig 1:**
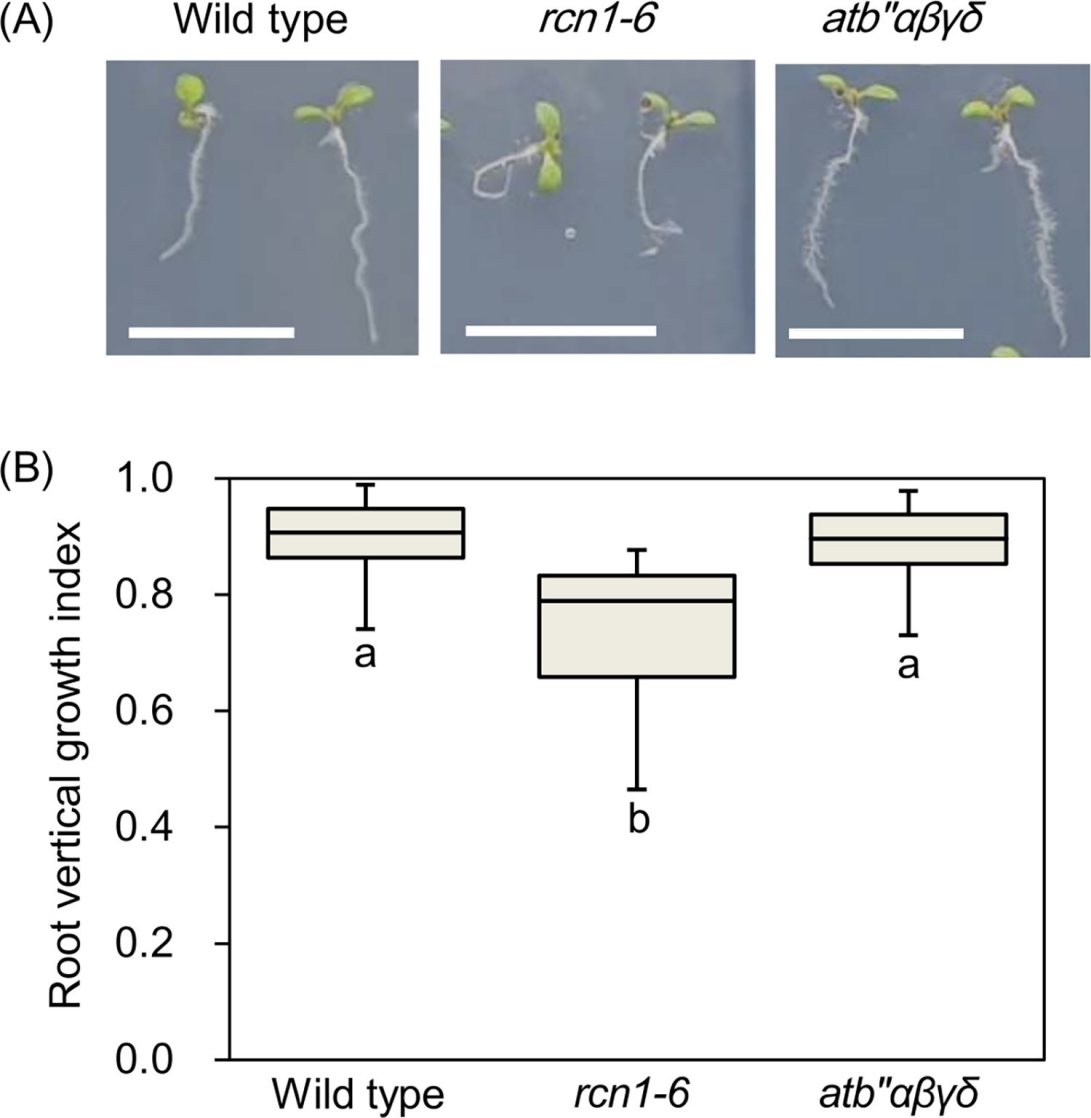
T-DNA insertions in *AtB’’α*, *AtB’’β*, *AtB’’γ*, and *AtB’’δ* do not affect touch-induced root bending. (A) Phenotypes of wild type, *rcn1-6*, and *atb’’αβγδ* seedlings grown on a 1.6% agar medium tilted at a 75° angle for 5 days. Representative images are presented for each genotype. Scale bars indicate 1 cm. (B) Root vertical growth indices (VGIs) of those seedlings. The top and bottom edges and the middle line of each box indicate quartiles, and the bar corresponds to the data range (*n* = 40 for each line). The data with different letters on them are different (*P* < 0.05) in the Dunnett’s test.

To examine functions of PP2A B’’ family subunits for touch-induced root bending, a mutant, *atb’’αβγδ*, which was generated from the mutants with T-DNA insertions in exons or introns of *AtB’’α*, *AtB’’β*, *AtB’’γ*, and *AtB’’δ* and which should thereby lack transcription or functions of these genes, was generated, and used in this study (S2A Fig). *rcn1-6* was also used (S2B Fig). Seedlings of wild type, *rcn1-6*, and *atb’’αβγδ* were germinated and vertically grown on mediums containing 1.6% agar. The primary root length of *atb’’αβγδ* seedlings were longer than that of wild-type and *rcn1-6* plants (S3 Fig left panel). The vertical projection length of roots was longer in the order of *atb’’αβγδ*, the wild type and *rcn1-6* (S3 Fig right panel). As a result, the vertical growth index (VGI) of *atb’’αβγδ* was similar to that of the wild type, and greater than that of *rcn1-6* (Fig 1).

**Fig 2:**
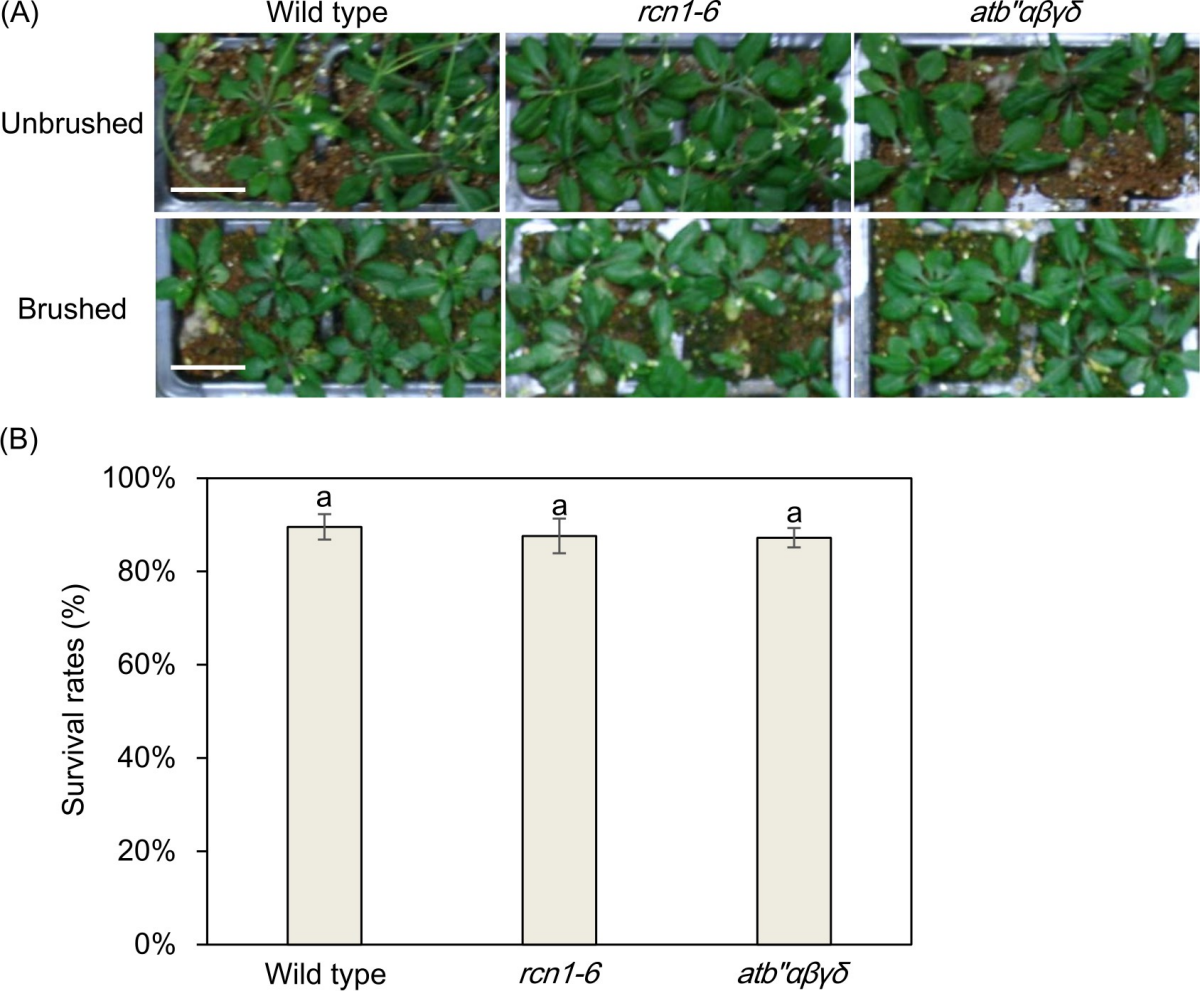
Leaves of the *atb’’αβγδ* plants under brushing-induced mechanical stress conditions. (A) The phenotypes of 28-day-old leaves from wild type, *rcn1-6*, and *atb’’αβγδ* plants grown in soil were observed after undergoing a brushing treatment. The pictures were taken seven days after the final brushing treatment. A representative image is presented, and a scale bar indicates 2 cm. (B) Survival rates of plants that underwent the brushing treatment. Values represent means ± SD of three biological replicates. Data with different letters are significantly different (P < 0.05) according to the Dunnett’s test.

To further examine the response to mechanical stress, these plants were subjected to continuous brushing to induce a strong mechanical stress that could cause cell death in mechanical stress-sensitive lines such as a VIP1-SRDX-overexpressing line [23]. No significant differences were observed in leaf phenotypes among *atb’’αβγδ*, *rcn1-6* and the wild type regardless of whether they were brushed or unbrushed (Fig 2).

### Differential expression of PP2A B’’ family subunits and VIP1/CAMTA-dependent induction of mechanical stress-responsive genes in *atb’’αβγδ*

RNA-Seq analysis was performed to investigate the transcriptomes of brushed leaves obtained from both wild type and *atb"αβγδ* plants. The 5’ region of *AtB"β* was not expressed in the *atb’’αβγδ* mutant, while the rest was overexpressed (S4 Fig). PR70, a human PP2A B’’ family subunit, has an FYF motif in the N-terminal regions; when this motif is truncated, PR70 cannot interact with PP2A A and C subunits [24]. AtB’’β has a similar FYY motif in its N-terminal region, which is absent in the *atb’’αβγδ* mutant (S4 Fig). This suggests that *AtB’’β* as well as *AtB"α*, *AtB"γ* and *AtB"δ* is not functional in the *atb’’αβγδ* mutant.

**Fig 3:**
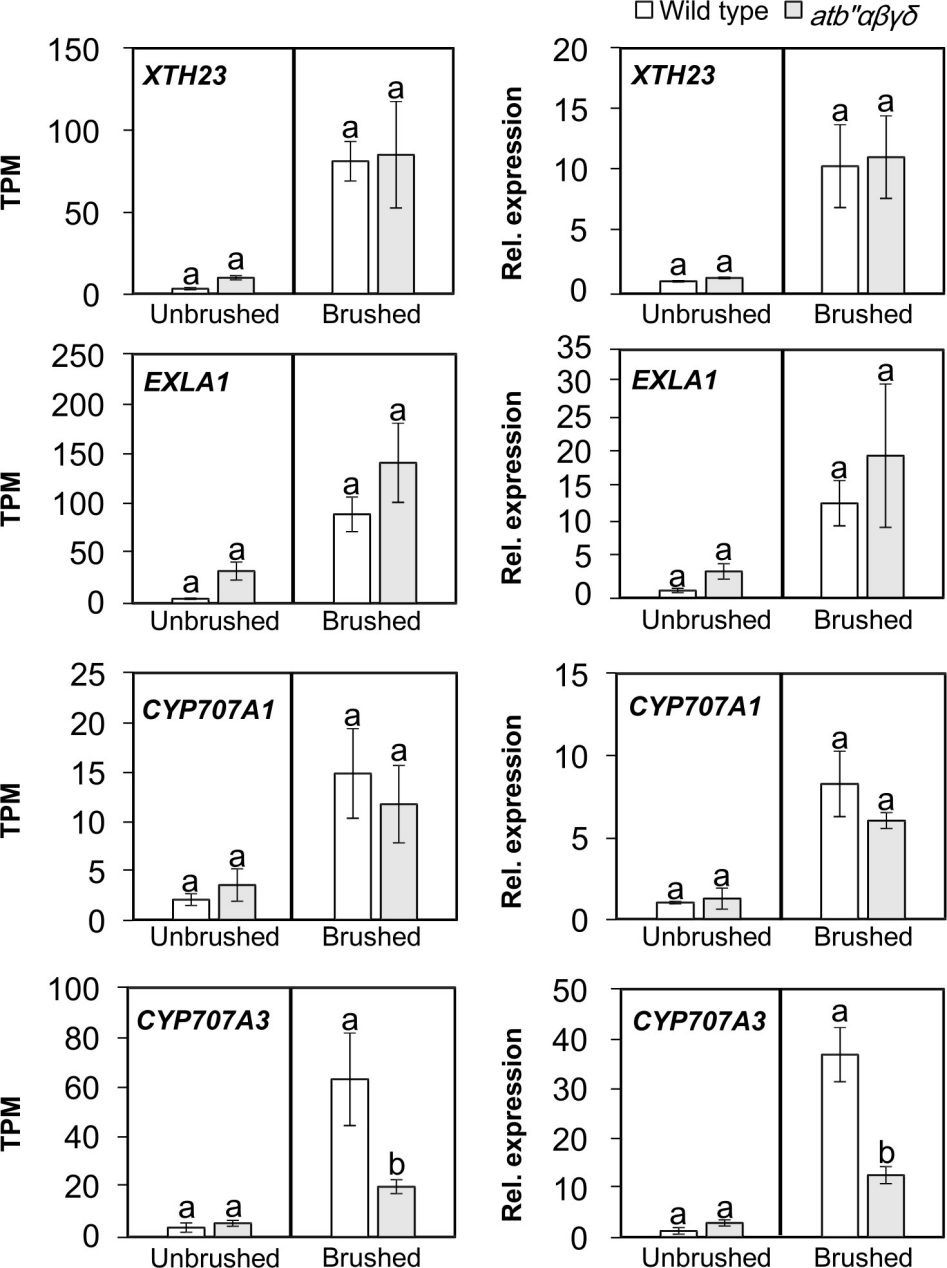
The expression levels of putative VIP1 target genes that were upregulated by brushing in brushed *atb’’αβγδ* leaves. TPM values were obtained from the RNA-Seq analysis, and the relative expression levels (’’Rel. expression’’) were calculated using qRT-PCR with the comparative cycle threshold (Ct) method with *UBQ5* as the internal control gene. The values presented are means ± SD from three biological replicates. No significant variation in Ct values of *UBQ5* between unbrushed and brushed samples (Ct values were 17.2 ± 0.2 for the unbrushed wild-type sample, 17.1 ± 0.2 for unbrushed *atb’’αβγδ*, 18.2 ± 0.7 for brushed wild-type, and 17.4 ± 0.3 for brushed *atb’’αβγδ*). Statistical tests were assessed by Holm-Sidak’s multiple comparison test when compared with the control sample.

Among the identified genes, two putative genes related to cell wall modification, *XYLOGLUCAN ENDOTRANSGLUCOSYLASE/HYDROLASE PROTEIN 23* (*XTH23*), and *EXPANSIN-LIKE ALPHA 1* (*EXLA1*), as well as two genes involved in abscisic acid (ABA) catabolism, *CYTOCHROME P450*, *FAMILY 707*, *SUBFAMILY A*, *POLYPEPTIDE1* (*CYP707A1*) and *CYP707A3*, contain the VIP1-binding element AGCTG(G/T) in their promoters [9] and can be upregulated by VIP1 within 20 min after hypo-osmotic stress that mimics mechanical stress [8, 10]. The expression levels of *XTH23*, *EXLA1*, and *CYP707A1* were similar between brushed *atb"αβγδ* leaves and wild type leaves in RNA-Seq (Fig 3, left panels) and qRT-PCR (Fig 3, right panels). However, the expression of the *CYP707A3* was significantly lower in brushed *atb"αβγδ* leaves compared to wild type leaves (Fig 3). These results suggest that the PP2A B" family B subunits are involved in regulating the expression of *CYP707A3*.

The brushing treatment resulted in a reduced number of upregulated and downregulated genes in *atb"αβγδ* leaves compared to wild type leaves (S5A Fig). In wild type, 3455 genes were upregulated and 1649 were downregulated after brushing, while in *atb"αβγδ*, 2059 genes were upregulated and 669 genes were downregulated (S5A Fig). A Venn diagram highlights the overlapping DEGs expressed in both wild type and *atb"αβγδ* after brushing (S5B Fig). The number of common genes that were upregulated and downregulated between wild type and *atb"αβγδ* was 1557 and 353, respectively (S5B Fig). The CAMTA-binding sequence, CGCG(C/T), was identified in a motif enriched in the promoters of genes upregulated by the brushing treatment in both wild type and *atb"αβγδ* plants (S5C Fig). These results are consistent with previous studies [25, 26].

**Fig 4:**
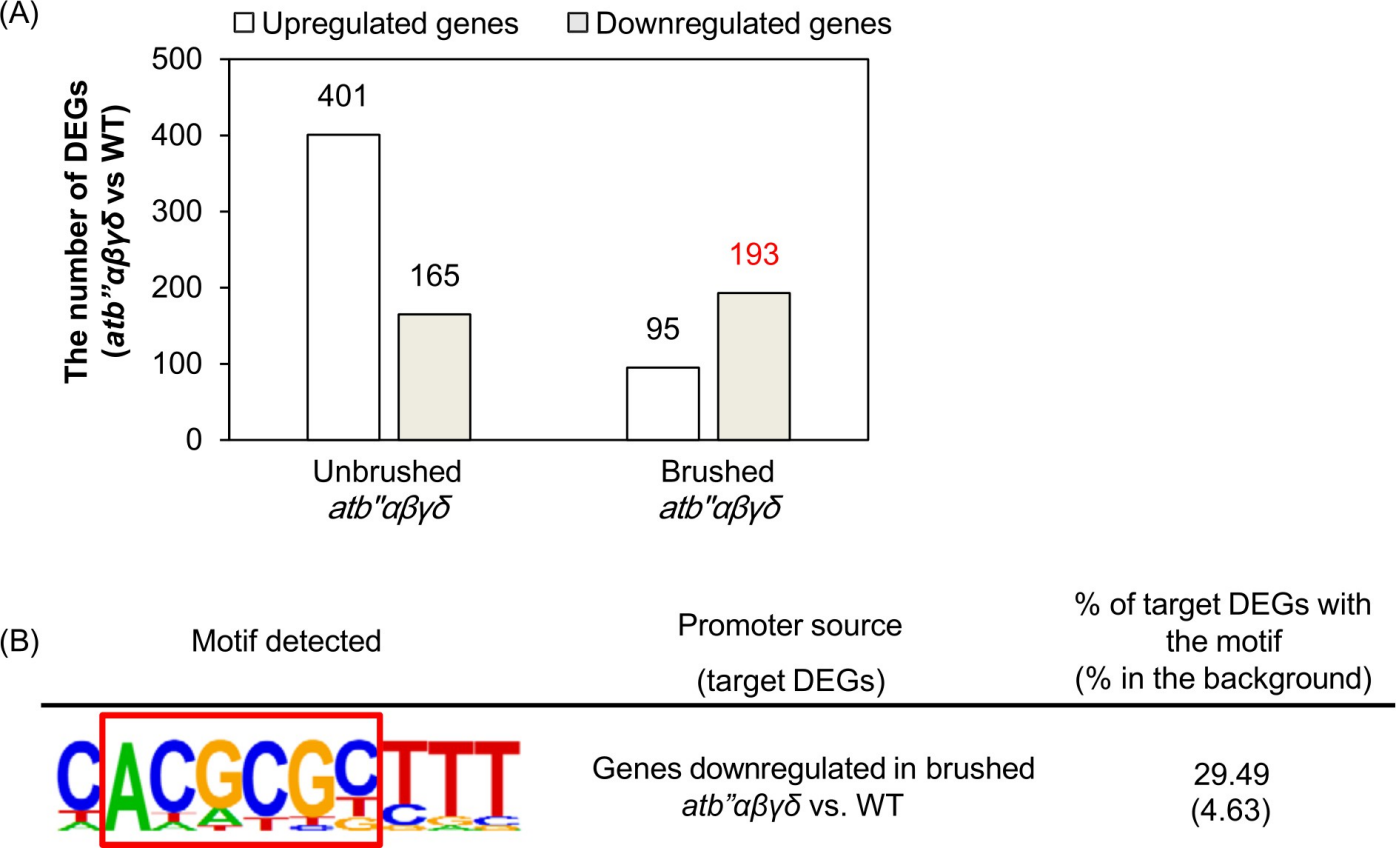
PP2A B’’ family subunits can regulate gene expression that contain CAMTA motifs (A) DEGs comprise genes that were either upregulated or downregulated in *atb’’αβγδ* compared to the wild type, with or without brushing. The determination of the number of DEGs involved a comparison of transcriptomes between *atb’’αβγδ* and wild type (WT), as detailed in the NCBI BioProject PRJNA899989. (B) The CAMTA-binding sequence, CGCG(C/T) was identified in the promoters of genes that exhibited weaker expression in *atb’’αβγδ* leaves compared to wild type leaves. The red box correspond to downregulated genes in “Brushed *atb’’αβγδ*” in panel A.

Unbrushed *atb’’αβγδ* leaves exhibited 401 upregulated genes and 165 downregulated genes compared to unbrushed wild type leaves (Fig 4A). Brushed *atb’’αβγδ* plants exhibited 95 upregulated genes and 193 downregulated genes compared to brushed wild type plants (Fig 4A). The promoters of genes downregulated in brushed *atb’’αβγδ* leaves compared to wild type leaves exhibited enrichment of the CAMTA-binding sequence, CGCG(C/T) (Fig 4B). These results raise the possibility that the PP2A B" family B subunits interact with and repress CAMTAs after mechanical stress.

### *atb’’αβγδ* mutants retard plant development under calcium deficiency and ABA after germination

**Fig 5:**
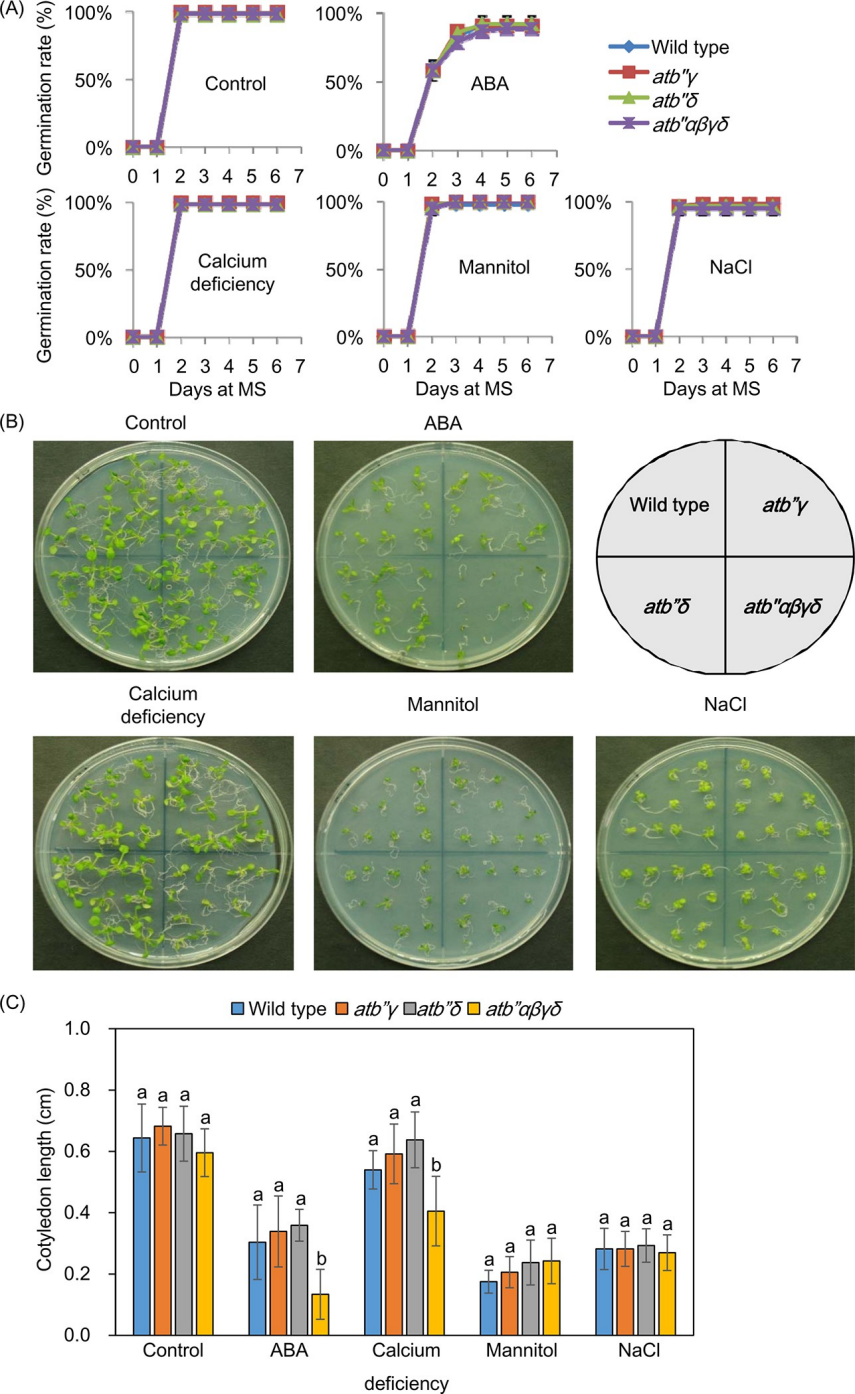
*atb’’αβγδ* plants exhibit retarded development under calcium-deficient and ABA-supplemented conditions after germination. (A) Germination rates of wild type, *atb’’γ*, *atb’’δ*, and *atb’’αβγδ* plants. They were germinated on different media, CaCl_2_-deficient medium, medium containing 100 mM NaCl, 200 mM mannitol, or 1 μM ABA. Germination rates were observed for 6 days. The presented data represents means ± SD of three replicates, each consisting of twenty plants per genotype. (B) and (C) Phenotypes and cotyledon length of 14-day-old seedlings were observed under the same various conditions. The data represents means ± SD (n = 10 for each genotype). Data with different letters indicate statistical significance (*P* < 0.05) based on Dunnett’s test.

Two *cyp707a3* knockout mutant lines, *cyp707a3-1* and *cyp707a3-2*, exhibits retarded growth rates under ABA-supplemented conditions after germination [27]. The germination rates of *atb’’αβγδ* plants were similar to wild type, *atb’’γ* and *atb’’δ* germination rates under ABA-supplemented, calcium-deficient, mannitol-stressed, NaCl-stressed, and unstressed conditions (Fig 5A). However, the cotyledon length of 14-day-old *atb’’αβγδ* plants were shorter than those of wild type, *atb’’γ* and *atb’’δ* plants under the ABA-supplemented and calcium-deficient conditions (Fig 5B and 5C). *atb’’αβγδ* plants also exhibited a lower greening rate under the ABA-supplemented conditions, whereas no significant difference was observed in greening rates between wild type, *atb’’αβγδ*, *atb’’γ* and *atb’’δ* plants under the other conditions (S6 Fig).

## Discussion

PP2A B’’ family subunits exhibit functional redundancy in VIP1-mediated gene expression under mechanical stress.

In this study, we show that PP2A B’’ family subunits, other than the specific PP2A B member, are responsible for the dephosphorylation of VIP1 (S1 Fig), suggesting that these subunits can specifically regulate the nuclear accumulation of VIP1. However, *atb’’αβγδ* plants exhibited wild type-like phenotypes in roots and leaves under various mechanical stress conditions (Figs 1 and 2). This indicates that the knockout of *AtB’’α*, *AtB’’β*, *AtB’’γ*, and *AtB’’δ* in the *atb’’αβγδ* plants is not sufficient to completely inhibit the dephosphorylation of VIP1 and its close homologs. Both AtB’’ε and FASS can interact with VIP1 [4], suggesting that these subunits should dephosphorylate VIP1 and its close homologs to induce mechanical stress responses even in the *atb’’αβγδ* mutant background. To test this possibility, future analyses with *AtB’’ε* and *FASS* mutants are necessary.

### PP2A B’’ family subunits play essential roles in plant development under calcium deficient and ABA-supplemented conditions

Transcriptome analysis showed a significant decrease in the expression of *CYP707A3*, which regulates ABA catabolism, in the *atb’’αβγδ* mutants (Fig 3). This raises the possibility that ABA accumulates in the *atb’’αβγδ* mutant background due to reduced *CYP707A3* gene expression, thereby potentially leading to the upregulation of ABA-regulated genes. Previous studies on other PP2A components indicated that a mutation in *RCN1* resulted in ABA hyposensitivity during seed germination and stomatal closure [28, 29]. Conversely, the PP2A catalytic C subunit mutant, *pp2ac2*, exhibited ABA hypersensitivity at various developmental stages [30]. Six members of the PP2A B’’ family subunits can be expressed in various tissues and interact with both PP2A A and C subunits [4]. Calcium promotes the formation of PP2A complexes dependent on PP2A B’’ family subunits [4]. Our results showed that the knockout of *AtB’’α*, *AtB’’β*, *AtB’’γ*, and *AtB’’δ* is sufficient to cause retarded growth under both ABA-supplemented and calcium-deficient conditions (Fig 5), even though *AtB’’ε* and *FASS* are still expressed in the *atb’’αβγδ* mutants (S1 Dataset). Our result may be similar case of the role of PP2A B′ family members, which regulate brassinosteroid-responsive gene expression and promote plant growth by interacting with and dephosphorylating BZR1 [31]. In this study, at least seven members of the PP2A B′ family can interact with BZR1. However, knocking out two of the nine PP2A B′ family members was sufficient to observe significant effects on plant brassinosteroid responses [31]. These findings suggest that knocking out four genes of the PP2A B’’ family subunits is sufficient to observe significant effects on plant responses to ABA or calcium-deficient conditions.

### PP2A B’’ family subunits can affect expression of CAMTA-dependent genes under mechanical stress

Calmodulin-binding transcription activators (CAMTAs) function as transcriptional repressors [25, 26]. Calcium-bound calmodulins can interact with and inhibit the transcriptional repression function of CAMTA3 and other CAMTAs, thereby dissociating CAMTAs from the CGCG(C/T) motifs in their promoters and activating defense-related genes and responses [26, 32]. Our motif analysis of DEGs from brushed and unbrushed samples showed that the CAMTA-binding sequence, CGCG(C/T) was enriched in the promoters of genes upregulated in both the *atb’’αβγδ* mutants and wild type (S5C Fig). However, further motif analysis of downregulated genes in brushed *atb’’αβγδ* mutants compared to brushed wild type revealed the presence of the CAMTA-binding sequence, CGCG(C/T) (Fig 4B). These findings raise the possibility that calcium-bound PP2A B’’ family subunits function similarly to calcium-bound calmodulins in repressing CAMTAs. *CAMTA1* and *CAMTA3* confer drought tolerance via regulating ABA response [33, 34]. Based on this idea, CAMTAs may be hyperactive in *atb’’αβγδ* mutants under brushed or ABA-supplemented conditions. Further study is required to elucidate interactions between PP2A B’’ family subunits and CAMTAs.

## Conclusion

This study highlights the distinct roles of PP2A B’’ family subunits in VIP1-mediated gene expression under mechanical stress and their importance in plant development under calcium-deficient and ABA-supplemented conditions. We suggest that PP2A B’’ family subunits influence *CYP707A3* expression and regulate CAMTA-dependent gene expression in response to mechanical stress. We also suggest that these subunits play a crucial role in plant development under conditions of ABA and calcium deficiency.

## Supporting information

S1 Fig PP2A B’’ family subunits are responsible for VIP1 dephosphorylation *in vitro*. (PDF)

S2 Fig T-DNA insertion mutants used in this study. (PDF)

S3 Fig Measurements of primary root length and its vertical projection for the wild type, *rcn1-6*, and *atb’’αβγδ*. (PDF)

S4 Fig Visualization of the positions of the reads mapped on *AtB’’β* in the *atb’’αβγδ* mutant. (PDF)

S5 Fig DEGs and motifs identified by RNA-seq with *atb”αβγδ* plants. (PDF)

S6 Fig Growth of *atb’’αβγδ* plants is retarded by ABA. (PDF)

S1 Table Primer pairs used for genotyping. (PDF)

S2 Table Primer pairs used for qRT-PCR. (PDF)

S1 Dataset All TPM values. (XLSX)

S2 Dataset All DEGs in wild-type and *atb’’αβγδ* plants. (XLSX)

S3 Dataset Homer-derived motifs. (7Z)
