# Supplementary material for: Characterizing the role of PP2A B’’ family subunits in mechanical stress response and plant development through calcium and ABA signaling in Arabidopsis thaliana
Source: PLoS One. 2024 Nov 14;19(11):e0313590. doi: 10.1371/journal.pone.0313590 (PMC11563394; doi:10.1371/journal.pone.0313590)
Supplement: S1 Fig — (PDF) [file pone.0313590.s001.pdf]

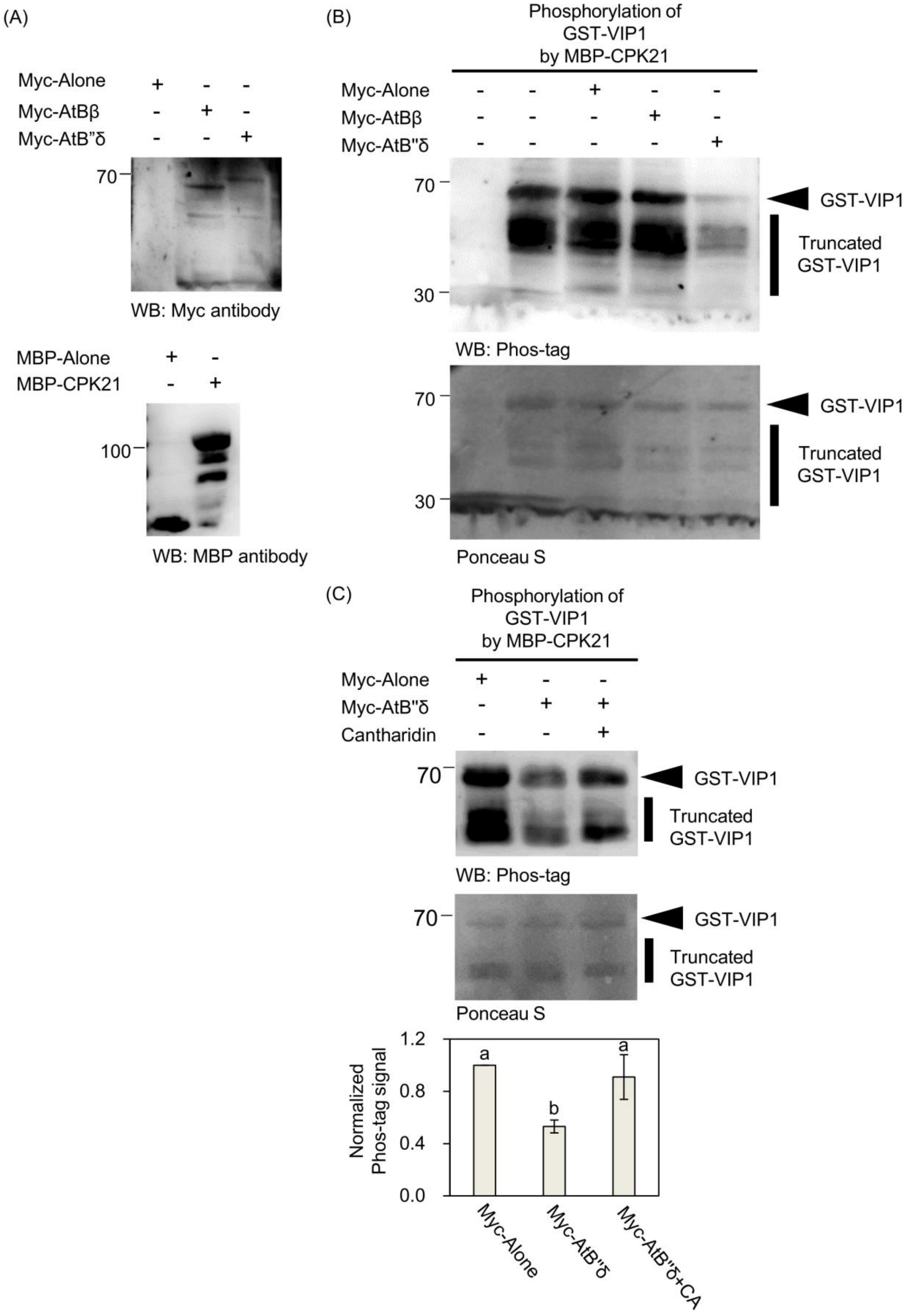
(A)
Myc-Alone + - -
Myc-AtBβ - + -
Myc-AtB"δ - - +
70
WB: Myc antibody
MBP-Alone + -
MBP-CPK21 - +
100
WB: MBP antibody
(B)
Phosphorylation of GST-VIP1 by MBP-CPK21
Myc-Alone - - + - -
Myc-AtBβ - - - + -
Myc-AtB"δ - - - - +
70
30
GST-VIP1
Truncated GST-VIP1
WB: Phos-tag
70
30
GST-VIP1
Truncated GST-VIP1
Ponceau S
(C)
Phosphorylation of GST-VIP1 by MBP-CPK21
Myc-Alone + - -
Myc-AtB"δ - + +
Cantharidin - - +
70
GST-VIP1
Truncated GST-VIP1
WB: Phos-tag
70
GST-VIP1
Truncated GST-VIP1
Ponceau S
Normalized Phos-tag signal
1.2
0.8
0.4
0.0
a
b
a
Myc-Alone
Myc-AtB"δ
Myc-AtB"δ+CA

Fig S1. PP2A B'' family subunits are responsible for VIP1 dephosphorylation *in vitro*. (A) Western blotting to examine the recombinant proteins used in the *in vitro* dephosphorylation assays. MBP (MBP-Alone) and MBP-tagged CPK21 (MBP-CPK21) were expressed in *Escherichia coli*, immobilized on the amylose resin, and detected using an anti-MBP antibody (WB: MBP antibody) on the western blot. Myc alone (Myc-Alone), Myc-fused AtBβ (Myc-AtBβ), and Myc-fused AtB''δ (Myc-AtB''δ) were expressed in the TNT Quick Master Mix solution, which is based on rabbit reticulocyte lysate. Immunoprecipitation was performed, and the proteins were detected using an anti-Myc antibody (WB: Myc antibody) on the western blot. The presence and absence of MBP-tagged proteins and Myc-tagged proteins are indicated as '+' and '-', respectively. (B) *In vitro* dephosphorylation of VIP1 with AtB''δ and AtBβ. GST-fused VIP1 (GST-VIP1) was immobilized on the glutathione resin and phosphorylated by MBP-CPK21. The resulting resin was mixed with a solution containing no insert, a Myc tag (Myc-Alone), Myc-tagged AtBβ (Myc-AtBβ), or Myc-tagged AtB''δ (Myc-AtB''δ) and incubated for one hour at room temperature. GST-VIP1 was eluted from the resin and analyzed by western blotting using Phos-tag biotin (WB: Phos-tag) and Ponceau S staining. The experiments were performed three times, and a representative result is shown. The protein molecular mass (kDa) is indicated on the left. The presence and absence of each protein are indicated as '+' and '-', respectively. (C) Repression of AtB''δ-dependent VIP1 dephosphorylation by the PP2A inhibitor, cantharidin (CA). Phosphorylated GST-VIP1 was reacted with Myc-AtB''δ in the presence and absence of 50 μM cantharidin. For the bottom panel, the levels of Phos-tag biotin-derived signals for GST-VIP1 incubated with Myc-AtB''δ in the absence or presence of cantharidin were normalized by the signals of GST-VIP1 incubated with Myc-Alone obtained on the membrane with Phos-tag biotin. Data are presented as means ± standard deviation (SD) from three replicates. The data with different letters on them are different ($P < 0.05$) in Holm-Sidak's multiple comparison tests.
