# Supplementary material for: Characterizing the role of PP2A B’’ family subunits in mechanical stress response and plant development through calcium and ABA signaling in Arabidopsis thaliana
Source: PLoS One. 2024 Nov 14;19(11):e0313590. doi: 10.1371/journal.pone.0313590 (PMC11563394; doi:10.1371/journal.pone.0313590)
Supplement: S2 Fig — (PDF) [file pone.0313590.s002.pdf]

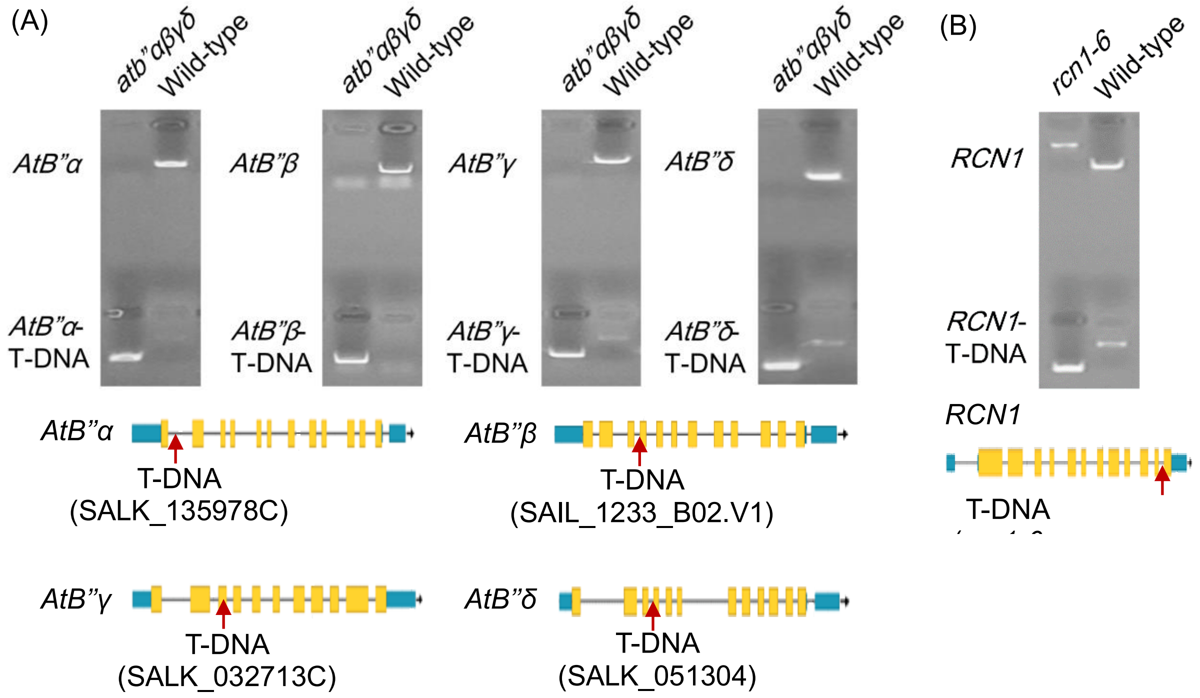


**Fig S2. T-DNA insertion mutants used in this study.** (A) Genomic PCR analyses of T-DNA insertions in the *AtB"α*, *AtB"β*, *AtB"γ*, and *AtB"δ* in *atb"αβγδ* plants. (B) T-DNA insertion in *RCN1* in *rcn1-6* plants. Representative results are presented for each target region and mutations in both A and B. In the bottom panels, untranslated regions (UTRs) and coding sequences (CDSs) in exons are indicated by blue and yellow boxes, respectively.
