# Supplementary material for: Characterizing the role of PP2A B’’ family subunits in mechanical stress response and plant development through calcium and ABA signaling in Arabidopsis thaliana
Source: PLoS One. 2024 Nov 14;19(11):e0313590. doi: 10.1371/journal.pone.0313590 (PMC11563394; doi:10.1371/journal.pone.0313590)
Supplement: S3 Fig — (PDF) [file pone.0313590.s003.pdf]

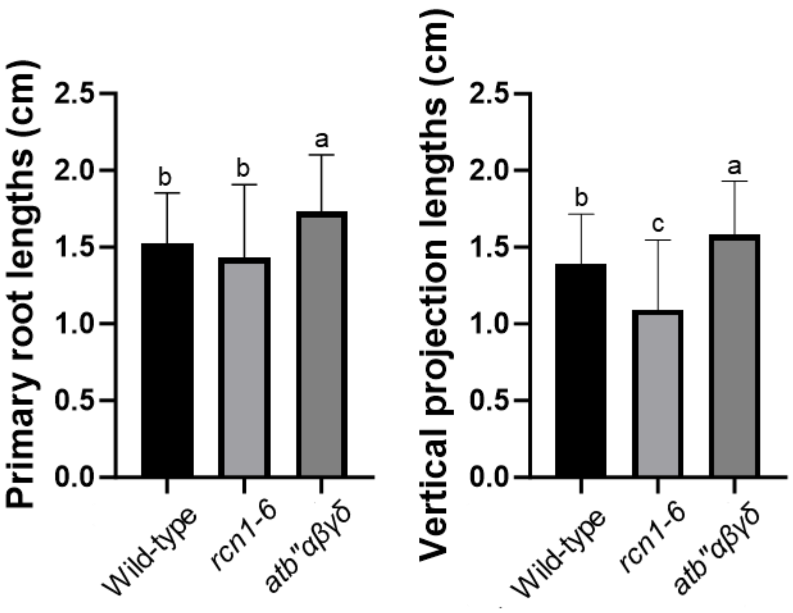

Fig S3. Measurements of primary root length and its vertical projection for the wild type, *rcn1-6*, and *atb''αβγδ*. The left panel shows the lengths of the primary roots, and the right panel shows the lengths of the vertical projections. These plants were grown for 5 days on agar medium, which was tilted at a 75° angle, and were used for the calculation of the VGIs (as shown in Fig. 1). The data are presented as means ± SD (n = 40 for each genotype). The data with different letters are significantly different ($P < 0.05$) according to the Dunnett's test.
