# Supplementary material for: Characterizing the role of PP2A B’’ family subunits in mechanical stress response and plant development through calcium and ABA signaling in Arabidopsis thaliana
Source: PLoS One. 2024 Nov 14;19(11):e0313590. doi: 10.1371/journal.pone.0313590 (PMC11563394; doi:10.1371/journal.pone.0313590)
Supplement: S4 Fig — (PDF) [file pone.0313590.s004.pdf]

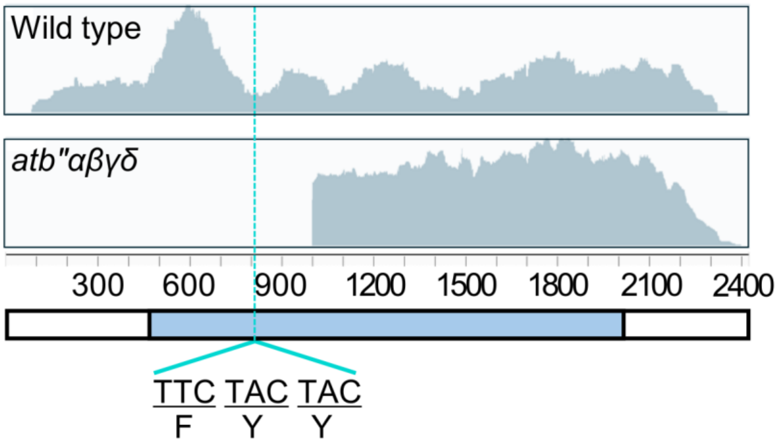

Fig S4. Visualization of the positions of the reads mapped on *AtB''β* in the *atb''αβγδ* mutant. Mapping coverages of the wild type- and *atb"αβγδ*-derived RNA-Seq reads are shown in the top and middle panels, respectively. The bottom panel shows the untranslated regions (white boxes), the coding sequence (light blue box) and the codons encoding the FYY motif of *AtB"β*.
