# Supplementary material for: Characterizing the role of PP2A B’’ family subunits in mechanical stress response and plant development through calcium and ABA signaling in Arabidopsis thaliana
Source: PLoS One. 2024 Nov 14;19(11):e0313590. doi: 10.1371/journal.pone.0313590 (PMC11563394; doi:10.1371/journal.pone.0313590)
Supplement: S5 Fig — (PDF) [file pone.0313590.s005.pdf]

(A)

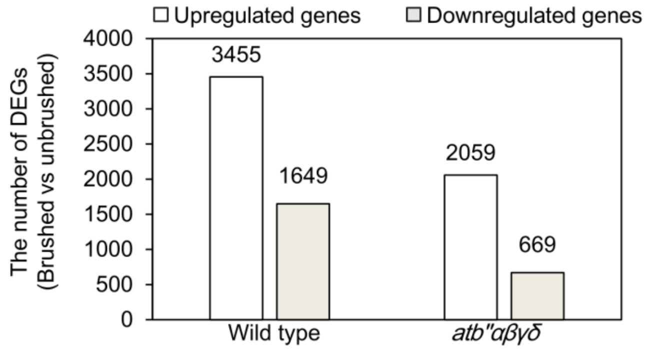

(B)

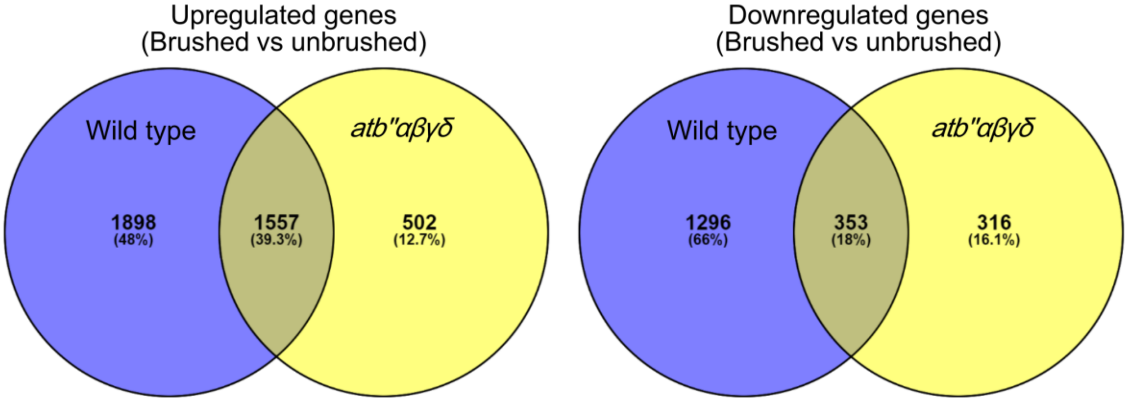

(C)

Motif detected	Promoter source (target DEGs)	% of target DEGs with the motif (% in the background)
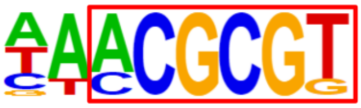	Genes upregulated in brushed WT vs. unbrushed WT	22.01 (7)
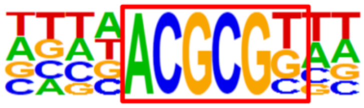	Genes upregulated in brushed *atb"αβγδ* vs. unbrushed *atb"αβγδ*	31.12 (6.23)

Fig S5. DEGs and motifs identified by RNA-Seq with *atb"αβγδ* plants. (A) The number of the DEGs identified in the brushed wild-type and *atb"αβγδ* plants. (B) Venn diagram showing an upregulated (left side) and downregulated (right side) genes between unbrushed samples and brushed samples. Venny 2.1.0 was used for generating Venn diagram (https://bioinfogp.cnb.csic.es/tools/venny/) (C) The motifs identified in the promoters of the upregulated genes in wild type (WT) and *atb"αβγδ* after brushing. The CAMTA-binding sequence CGCG(C/T) is highlighted in the red box.
