# Supplementary material for: Characterizing the role of PP2A B’’ family subunits in mechanical stress response and plant development through calcium and ABA signaling in Arabidopsis thaliana
Source: PLoS One. 2024 Nov 14;19(11):e0313590. doi: 10.1371/journal.pone.0313590 (PMC11563394; doi:10.1371/journal.pone.0313590)
Supplement: S6 Fig — (PDF) [file pone.0313590.s006.pdf]

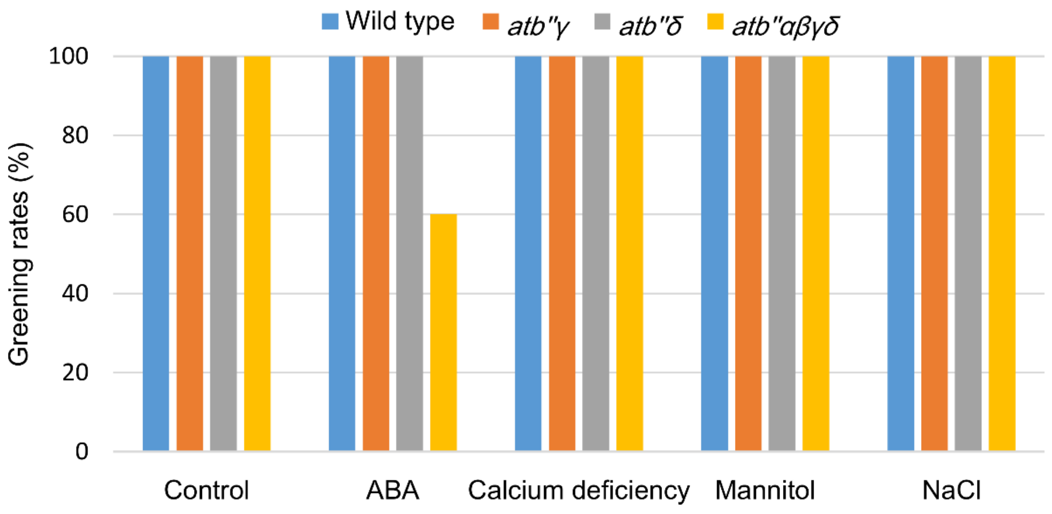


**Fig S6. Growth of *atb''αβγδ* plants is retarded by ABA.** For each genotype, 10 plants were assessed for the presence of green seedlings when they were 14 days old.
