# Supplementary material for: Characterizing the role of PP2A B’’ family subunits in mechanical stress response and plant development through calcium and ABA signaling in Arabidopsis thaliana
Source: PLoS One. 2024 Nov 14;19(11):e0313590. doi: 10.1371/journal.pone.0313590 (PMC11563394; doi:10.1371/journal.pone.0313590)
Supplement: S1 Table — (PDF) [file pone.0313590.s007.pdf]

Table S1. Primer pairs used for genotyping.

Primers	Sequence (5'>3')
gDNA bppa(670)_Fw	GCTTCCCGGTTTGTCTCCTG
gDNA bppa(1600)_Rv	CAACTGTCAAGCAAGGTAGTGC
atbppg_gDNA(502)Fw	CCGGACGGTAACACAACGAA
atbppg-gDNARv	CATGCTGCATTGCGTCAACTA
atbppd_gDNA(1019)Fw	TGACAATGATGCCTTAGGTGGT
atbppd_gDNA(2300)Rv	AGTACATCATACCAAAGTGGCA
gDNA rcn1-6_Fw	GTCAAGTACCTCACCTATAAC
gDNA rcn1-6_Rv	CATAGCCAGCAACCAAAATGG
T-DNA_LBTR3	CAGCTGTTGCCCGTCTCACTGG
PP2ABppb-2g_F	TCATTTCCTGCCTCTGGACC
PP2ABppb-2g_R	ACTGGTTTGAAGTCTGCCTGT
pDAP101LB1	GCCTTTTCAGAAATGGATAAATAGCCTTGCTTCC
