# Supplementary material for: Characterizing the role of PP2A B’’ family subunits in mechanical stress response and plant development through calcium and ABA signaling in Arabidopsis thaliana
Source: PLoS One. 2024 Nov 14;19(11):e0313590. doi: 10.1371/journal.pone.0313590 (PMC11563394; doi:10.1371/journal.pone.0313590)
Supplement: S2 Table — (PDF) [file pone.0313590.s008.pdf]

Table S2. Primer pairs used for qRT-PCR

Genes		Sequence (5'>3')
EXLA1	Fw	CCACCGGAGCTATCCAGTTC
	Rv	AAGATGTTTGTTTGTGTGCCACT
XTH23	Fw	TGGTTCGTGGTTGTCTCAGG
	Rv	CTAAGCACTCGCGTGGAAGA
CYP707A1	Fw	TGGAACCCACTCGTGTCCTGGA
	Rv	CCCGTCGCTCGCTCCAACAA
CYP707A3	Fw	TCGAAGTTGCGCCGAAACCGA
	Rv	GGCCCTACGATTGACCATCTGTACT
UBQ5	Fw	GACGCTTCATCTCGTCC
	Rv	CCACAGGTTGCGTTAG
